# Malaria Resurgence in the Americas: An Underestimated Threat

**DOI:** 10.3390/pathogens8010011

**Published:** 2019-01-18

**Authors:** J. Luis Espinoza

**Affiliations:** Department of Hematology and Rheumatology, Kindai University Faculty of Medicine, Osaka-Sayama, Osaka 577-8502, Japan; luis@med.kindai.ac.jp; Tel.: +81-72-366-0221

Malaria is a mosquito-borne disease caused by parasites of the genus *Plasmodium* (*P. falciparum*, *P. vivax*, *P. ovale*, *P. malariae*, and *P. knowlesi*) and acquired through the bites of infected Anopheles mosquitos. With an incubation period of 10 to 15 days after the infective mosquito bite, the disease manifests with fever, chills, headaches, vomiting and tiredness. Although the disease is preventable and treatable, untreated malaria is typically associated with anemia and splenomegaly. In particular, *P. falciparum* cases commonly progress to life-threatening conditions including “cerebral malaria”, organ dysfunction and death [1].

Hippocrates was the first to systematically describe the typical periodic recurrent fever in patients with malaria and molecular studies have documented traces of this disease in tissues from ancient Egyptian mummies, indicating that years before Alphonse Laveran in 1880 discovered the cause of malaria, clinicians have had to deal with this devastating disease [2]. Nowadays, malaria has been virtually eradicated from technologically advanced countries. However, by the end of the 20th century, malaria was a neglected disease causative of more than one million deaths per year. According to the World Health Organization (WHO) estimates, between 2000 and 2015 (a period when large-scale malaria control interventions were implemented), global malaria incidence decreased by one third and mortality rates declined by more than half (*World Malaria Report 2016, WHO, Geneva*).

Despite the above-described achievements, malaria is still endemic in many countries around the world. Millions of people do not have access to malaria commodities (such as antimalarial drugs, insecticides, and diagnostic supplies) and thousands of malaria cases remain unreported [1], indicating that worldwide disease control measures are still insufficient. Children under 5 years of age are especially vulnerable to this disease, with nearly 70% of all malaria deaths occurring in this group. This figure means that every hour, 30 children die of malaria. In the United States, more than 1700 cases of malaria are diagnosed each year, the majority being travelers and immigrants returning from countries where malaria transmission occurs. Most cases could be prevented by following prevention recommendations, including antimalarial chemoprophylactic regimens and using mosquito bite prevention measures [3].

According to latest WHO malaria report, released in November 2018, the estimated incidence rate in 2017 was 219 million cases worldwide (95% confidence interval (CI): 203–262 million), with most cases in the African region (200 million or 92%). In comparison with data from 2010, there was an estimate of 20 million fewer malaria cases in 2017, but this figure is up from the 217 million cases reported in 2016. Last year there was an estimated 435,000 malaria deaths worldwide, a decrease of about 10,000 deaths over the previous year. Of note—apart from the WHO region of the Americas, where malaria mortality actually increased—in all WHO regions, malaria mortality either declined or leveled off (*World Malaria Report 2018, WHO, Geneva*).

The increased malaria mortality in the Americas is largely due to disease resurgence in Venezuela and to a lesser extent in Nicaragua, Brazil, and Peru (Figure 1). By 1931, Venezuela had the highest malaria incidence rate in Latin America, but intensive malaria control efforts allowed this nation to be recognized in 1961 by the WHO as the first country in the world to eliminate malaria in its most populated area [4,5]. Such an astonishing achievement has been overshadowed by an unprecedented resurgence of the disease. Malaria in Venezuela is out of control. Between 2000 and 2015, cases increased by 365% and in 2017 alone, Venezuela accounted for 53% of all confirmed and presumed cases of malaria in the Americas (*World Malaria Report 2018, WHO, Geneva*).

A severe shortage of first-line antimalarial drugs, scarce investment in vector control measures, loss of equipment and infrastructure, and a collapsed health system as a result of the ongoing political and economic crisis (annual inflation rate of 1,000,000%), are major determinants of the malaria resurgence in Venezuela [4,5]. In addition, illegal mining activities in areas with high malaria transmission, especially in the state of Bolivar, are contributing to the aggravation of the malaria situation in Venezuela [4]. Meanwhile, malaria cases in Venezuela have spilled over to other neighboring countries in the region, overloading local health care services in Brazil and Colombia [4]. The large-scale migration of Venezuelan nationals, aggravated in the last six months, is a reasonable concern that the growing malaria epidemic in Venezuela could spread to the entire region.

Malaria cases in Nicaragua have steadily increased from 2010 and, just in 2017, cases jumped more than 1400% compared to 2010, which has been attributed to changes in internal migration and socio-economic and environmental factors that have overwhelmed the local response to malaria infective mosquitoes in the country’s Caribbean region, where most cases have been reported (*World Malaria Report 2018, WHO, Geneva*). Nevertheless, the ongoing social unrest and political crisis that exploded in April 2018 will likely worsen this situation since financial constraints derived from the economic crisis as well as the reported loss of trained human resources, including those involved in malaria control programs [6], will further compromise the efficacy of the country’s malaria control program. 

Blood transfusion-transmitted malaria is another concern in the Americas. More than 400 cases were documented in the region between 1971 and 2016, with 50% reported in Mexico and 40% in the USA [7]. This underscores the importance of asymptomatic infections for disease spread. Notably, microscopic analysis of blood smears remains the standard method for malaria diagnosis in Latin America [4], which requires highly trained technicians to identify parasite morphology. This method also has low sensitivity to detect cases with low parasitemia and is unavailable in remote areas without microscopy facilities, emphasizing the importance of introducing rapid diagnostic tests and other molecular diagnostic tools to improve the diagnosis of the disease.

Artemisinin-resistant *P. Falciparum* parasites were first reported in Southeast Asia 10 years ago [8] and, although no cases of artemisinin resistance have been reported in the Americas, the potential emergence of artemisinin-resistant parasites in the region is a major concern. Importantly, recent studies have shown that bone marrow tissues are a major reservoir of infective *P. vivax* [9] (the most frequent agent, causative of most malaria cases in the Americas), and recent animals studies suggest that *Plasmodium* parasites can evade antimalarial drugs in hematopoietic tissues where they can eventually develop drug resistance [10]. The administration of suboptimal antimalarial regimens in patients with malaria, especially in the setting of drug shortage as has been increasingly reported in Venezuela [4,5], may favor the emergence of drug-resistant parasites in such a hidden parasite reservoir.

The malaria situation in the Americas highlights the contributing effects of political and economic instability in the re-emergence of previously controlled transmissible diseases. The critical situation in Venezuela calls for urgent attention. This implies the need for regional collaboration initiatives, especially among Venezuela’s neighbors, to control malaria spread toward the rest of the region. Nevertheless, without the commitment and political willingness of the government of Venezuela, any effort will be insufficient. However, Maduro’s administration has repeatedly denied the existence of a humanitarian crisis in Venezuela and consequently has failed to call for an emergency plan to control malaria epidemics, limiting the participation and cooperation of foreign governments in malaria control measures. The recent inclusion of Venezuela, along with Nigeria, South Sudan, and Yemen, in the list of countries that require malaria emergency responses to deal with serious health risks (*WHO Malaria Report, Geneva; 2018*) will surely benefit the country through a coordinated global response to control this epidemic; however, other actions are needed.

## Figures and Tables

**Figure 1:**
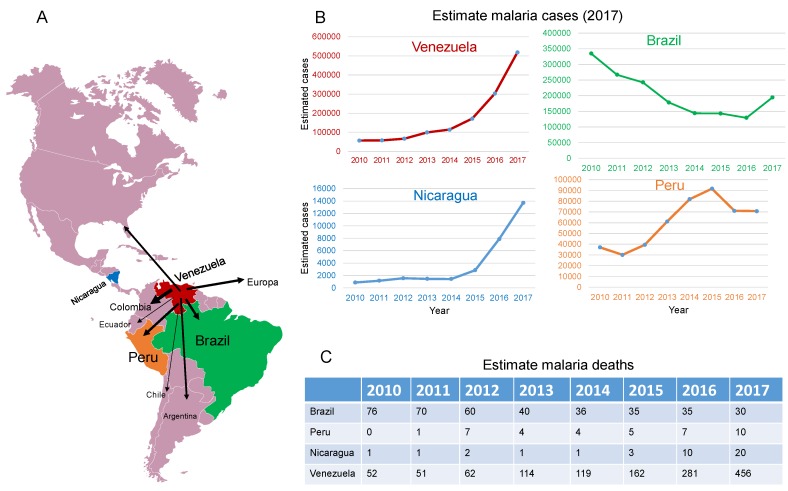
(**A**) Highlighted are the four countries (Brazil, Peru, Nicaragua, and Venezuela) where malaria cases increased significantly in 2017. Arrows indicate countries to which Venezuelan nationals are immigrating. (**B**) Estimated annual malaria cases in each country between 2010 to 2017. (**C**) Estimate malaria deaths in the same period. Source: World Health Organization (WHO) Malaria Report 2018. *World Malaria Report, WHO, Geneva; 2018* (Annexes 3F and 3H) https://www.who.int/malaria/publications/world-malaria-report-2018/en/).
